# Clinical and Coronary Angiographic Insights Into Acute ST-Elevation Myocardial Infarction in Young Patients: A Study From a Tertiary Care Cardiac ICU

**DOI:** 10.7759/cureus.68484

**Published:** 2024-09-02

**Authors:** Nitin Ramanathan, Hisham Mohammed Babu, Aparnna Nair, Dhivya Mohan, Md Abdur Rob, Raju Kumar Dey, Rishabh Jain, Naveenkumar Nallathambi, Aravind Muthiah, Tejaswee Lohakare, Tanisha Rathi

**Affiliations:** 1 Internal Medicine, Jagadguru Sri Shivarathreeshwara (JSS) Academy of Higher Education and Research, Mysore, IND; 2 Internal Medicine, Altnagelvin Hospital, Londonderry, GBR; 3 Internal Medicine, Aarupadai Veedu Medical College & Hospital, Pondicherry, IND; 4 Critical Care, East Lancashire Hospitals NHS Trust, Blackburn, GBR; 5 Orthopedics, University Hospitals Sussex NHS Foundation Trust, Brighton and Hove, GBR; 6 Critical Care, Cumberland Infirmary, North Cumbria Integrated Care NHS Foundation Trust, Carlisle, GBR; 7 Internal Medicine, Madras Medical College, Chennai, IND; 8 Internal Medicine, Tirunelveli Medical College, Tirunelveli, IND; 9 Child Health Nursing, Shrimati Radhikabai Meghe Memorial College of Nursing, Datta Meghe Institute of Higher Education & Research, Wardha, IND; 10 Medicine, Mahatma Gandhi Institute of Medical Sciences, Wardha, IND

**Keywords:** public health, clinical presentation, angiography, st elevation myocardial infarction (stemi), coronary artery disease (cad)

## Abstract

Background

This investigation addresses the major effects of early-onset coronary artery disease (CAD) on community health, noting its association with elevated incidences of recurrent ischemic events and mortality. The study specifically explores the contributing factors, clinical symptoms, angiographic findings, and management strategies for individuals aged 45 or younger who experience their initial ST-elevation myocardial infarction (STEMI).

Methodology

This observational study took place over a six-month period within the cardiology unit, involving 100 sequential patients diagnosed with STEMI.

Results

With a mean age of 42.5 years, the research had 100 patients, of which nine (9%) were under 25, 24 (24%) were between 26 and 35, and 67 (67%) were over 36. Of these, 89 (89%) were male. The following risk variables were found: obstructive CAD, smoking, being overweight, diabetes, hypertension, chest discomfort, and syncope. In 99 patients (99%), the most prevalent symptom was chest discomfort. Most often impacted was the left anterior descending (LAD) artery in 24 patients (24%), then the right coronary artery in 14 patients (14%). A total of 50 patients (50%) had percutaneous coronary intervention (PCI), with 15 patients (15%) undergoing elective PCI, 10 patients (10%) with pharmaco-invasive PCI, and 20 patients (20%) receiving primary PCI. In eight cases (8%), coronary artery bypass grafting (CABG) was required. Furthermore, 40 patients (40%) were under medical care, and 32 patients (32%) had recanalized and normal coronaries. No mortality was recorded in this study.

Conclusions

Acute myocardial infarction is most frequently seen in very young adult males, and the most common risk factor is smoking. The most common clinical manifestation, anterior wall myocardial infarction, was caused by the main source of involvement, the LAD artery.

## Introduction

Plaque accumulation in the coronary arteries leads to the common cardiac ailment known as coronary artery disease (CAD) [1]. This illness may start in childhood and get worse throughout the course of a person’s life. The estimated average age of the first acute myocardial infarction (AMI) in the United States is 72 years for women and 65.6 years for men, according to recent research by the American Heart Association [2]. Public health is greatly impacted by early-stage CAD because of its high risk of ischemia recurrence and death [1,2]. For this reason, research on CAD in youth is essential for both early diagnosis and therapeutic intervention.

While the exact definition of “young CAD” varies, it is generally considered to affect individuals aged 45-55 years. Previously, the age group associated with young CAD was 65, but more recent reports define premature CAD in men as occurring at 49 years. In young Indians, the prevalence of CAD is about 12-16%, higher than in any other ethnic group [3,4]. Studies suggest that 4-10% of AMI cases occur before the age of 45, although this frequency decreases with a lower age limit. Recently, young individuals have shown a higher risk of ST-elevation myocardial infarction (STEMI) [5].

The condition referred to as myocardial infarction with nonsignificant coronary artery obstruction (MINOCA) affects 10-20% of juvenile AMI patients. Important diagnostic and therapeutic instruments include cardiac and intravascular magnetic resonance imaging [6-8]. Pregnant and postpartum women are more likely to get MINOCA and spontaneous coronary artery dissection (SCAD). Furthermore, younger individuals are more likely to experience vasospastic (Prinzmetal) angina, which has a different prognosis and course of therapy than obstructive CAD [6,7,9].

There is a lack of clinical angiography studies on young males suffering from STEMI. Therefore, studying the clinical and angiographic profiles of young males (under 45 years of age) experiencing acute STEMI is essential for better identification and preventive measures. The main objective is to examine the risk factors, angiographic profiles, clinical presentations, and management strategies for very young adults (aged ≤45 years) experiencing their first AMI.

## Materials and methods

Study design and setting

This cross-sectional study was conducted at Jagadguru Sri Shivarathreeshwara (JSS) Academy of Higher Education and Research in Mysore, India, involving 100 consecutive patients diagnosed with STEMI over six months in the cardiology department. The study received prior approval from the institutional ethics committee.

Selection Criteria

The selection of research participants was made in accordance with the European Society of Cardiology’s standards for assessing whether an ECG suggests a STEMI. The new V2-V3 contiguous ST-segment elevation had to be at least 2 mm (1.5 mm for women and 2 mm for men) at the J point, and it had to be at least 1 mm in another neighboring chest or limb leads. In order to participate, one must be younger than 45 and free of the left bundle branch block. Exclusion criteria included a history of percutaneous coronary intervention (PCI), bundle branch block, myocardial infarction, electrolyte imbalances, prior coronary artery bypass graft (CABG) surgery, or left ventricular hypertrophy as determined by an ECG. Additionally, patients over 45 years old, and those with conditions affecting the ST segment (such as suspected myocarditis, pericarditis, hypothermia, or those on amiodarone therapy) were excluded.

Data sources and variables

Each patient underwent a thorough clinical evaluation, including a detailed family history and standard biochemical tests like complete blood count, urea, creatinine, and viral markers (HIV, hepatitis C virus, and hepatitis B surface antigen). Additional tests included an echocardiogram, ECG, fasting lipid profile, postprandial blood sugar, and fasting blood sugar. Risk factors such as smoking, obesity, hypertension, diabetes mellitus (DM), physical inactivity, hyperlipidemia, and early CAD (first-degree relatives under 55 years in men and under 65 years in women) were assessed. Smokers were categorized into those who had quit smoking within the last year and those currently smoking for over four weeks.

Clinical and Anthropometric Assessments

Clinical evaluations and physical measurements were performed, including monitoring of blood pressure (BP). Participants donned light attire and went barefoot for their height and weight assessments. Weight was taken to the nearest 0.1 kg with a precise digital scale, and height was recorded to the nearest 5 mm with a stadiometer. The BMI was determined using Quetelet’s formula: BMI = mass (kg)/height (m)².

A BMI over 25 kg/m² was deemed overweight. The patient was in a supine posture when the BP was taken with an appropriate-sized cuff using a sphygmomanometer on the left arm. High BP was classified as having a systolic measurement of at least 140 mmHg or a diastolic measurement of 90 mmHg or higher. DM was diagnosed if the patient had a history of diabetes, a fasting plasma glucose level of 126 mg/dl or above, a postprandial plasma glucose level of 200 mg/dl or higher, or was taking antidiabetic medications. Hyperlipidemia was identified based on a history of treated dyslipidemia, serum cholesterol levels of 200 mg/dl or more, triglycerides (TG) levels of 150 mg/dl or higher, low-density lipoprotein (LDL) levels of 130 mg/dl or more, high-density lipoprotein (HDL) levels below 50 mg/dl for women and below 40 mg/dl for men, or any combination of these criteria.

Most patients received fibrinolysis therapy with 1.5 million units of streptase, except those undergoing primary coronary angiography. All patients underwent coronary angiography. Obstructive CAD was characterized by a ≥50% blockage in the left main coronary artery or a ≥70% blockage in the principal epicardial arteries. Stenosis in major epicardial arteries ranging from 50% to 69% was classified as an intermediate disease, while lesions ≤50% were considered a minor disease. Both intermediate and minor diseases were combined and classified as nonobstructive diseases. Patients were treated with PCI, medical therapy, or CABG after identifying the culprit artery through angiographic results.

Statistical analysis

Microsoft Excel (Microsoft Corporation, Redmond, WA, USA) was used to enter the data into a master graphic, and IBM SPSS Statistics for Windows, Version 20.0 (Released 2011; IBM Corp., Armonk, NY, USA) was used for analysis. Categorical and continuous variables were described using descriptive statistics, such as mean, percentage, and frequency analyses. Data from statistics were shown as mean ± SD.

## Results

Table 1 provides a detailed summary of the demographic and clinical profiles of 100 patients diagnosed with STEMI. A significant majority of the patients were older than 36 years (67 patients, 67%) and male (89 patients, 89%). Common risk factors identified include smoking (81 patients, 81%), hypertension (29 patients, 29%), and DM (40 patients, 40%). In terms of BMI, most patients were categorized as having a normal weight (81 patients, 81%). The predominant symptoms experienced by patients were chest pain (99 patients, 99%), sweating (70 patients, 70%), and breathlessness (64 patients, 64%). Clinically, the most frequent diagnosis was anterior wall myocardial infarction (AWMI; 60 patients, 60%), followed by inferior wall myocardial infarction (39 patients, 39%).

**Table 1:** Demographic and clinical characteristics of 100 STEMI patients AWMI, anterior wall myocardial infarction; CAD, coronary artery disease; DM, diabetes mellitus; HTN, hypertension; MI, myocardial infarction; STEMI, ST-elevation myocardial infarction

Variables	Count (N)	Percentage (%)
Age group (years)		
<25	9	9%
26-35	24	24%
>36	67	67%
Gender		
Female	11	11%
Male	89	89%
Risk factors		
Smoking	81	81%
HTN	29	29%
DM	40	40%
Family history of CAD	18	18%
BMI		
Underweight	1	1%
Normal weight	81	81%
Overweight	18	18%
Types of symptoms		
Chest pain	99	99%
Diaphoresis (sweating)	70	70%
Dyspnea (breathlessness)	64	64%
Nausea/vomiting	52	52%
Palpitations	40	40%
Diarrhea	18	18%
Syncope (fainting)	13	13%
Clinical STEMI diagnosis		
AWMI	60	60%
Inferior wall MI	39	39%
Inferior wall MI with right ventricular MI	4	4%
Lateral wall MI	3	3%

Table 2 shows the lipid profile analysis of the patients. The mean total cholesterol (TC) level was 210.5 mg/dL with an SD of 35.50 mg/dL. The LDL had a mean value of 125.8 mg/dL and an SD of 20.40 mg/dL. The TGs were found to have a mean of 195.3 mg/dL and an SD of 45.80 mg/dL. Lastly, the HDL showed a mean level of 42.1 mg/dL with a standard deviation of 6.20 mg/dL.

**Table 2:** Lipid profile of patients HDL, high-density lipoprotein; LDL, low-density lipoprotein; TC, total cholesterol; TG, triglycerides

Lipid profile component	Mean ± SD (mg/dL)
TC	210.5 ± 35.50
LDL	125.8 ± 20.40
TG	195.3 ± 45.80
HDL	42.1 ± 6.20

Table 3 details the distribution of obstructive CAD among 100 patients. The left anterior descending (LAD) artery emerged as the most commonly affected region, involving 24 patients (24%). The right coronary artery (RCA) was the second most frequently impacted, affecting 14 patients (14%) of the cohort. Triple vessel disease (TVD) was identified in 12 patients (12%). Instances involving the left circumflex (LCX) and ostial LAD each accounted for four patients (4%), while less common combinations such as RCA and LAD and RCA and LCX were observed in smaller percentages. Additionally, left main (LM) disease affected five patients (5%), and a diverse group of other multi-vessel diseases was seen in 21 patients (21%) of the cases.

**Table 3:** Distribution of patients with obstructive CAD in a sample of 100 patients CAD, coronary artery disease; LAD, left anterior descending; LCX, left circumflex; LM, left main; RCA, right coronary artery; TVD, triple vessel disease

Affected artery/region	Number of patients (N)	Percentage (%)
LAD	24	24%
LCX	4	4%
Ostial LAD	4	4%
Ostial RCA	2	2%
RCA	14	14%
LAD and diagonal branch (D1)	2	2%
LAD and LCX	2	2%
RCA and LAD	3	3%
RCA and LCX	4	4%
TVD	12	12%
LM disease	5	5%
Combined LAD, RCA, and LCX	3	3%
Multi-vessel disease (other)	21	21%

Table 4 summarizes the coronary angiography findings of 100 patients. Among the lesion types, Type A lesions were the most prevalent, affecting 35 patients (35%), followed by Type B and Type C lesions, seen in 15 patients (15%) and 10 patients (10%) of the cohort, respectively. The majority of patients, 80 patients (80%), had no thrombus (Grade 0), while thrombus grades 2, 3, 4, and 5 were less common, collectively affecting 20 patients (20%). Mild calcification was observed in seven patients (7%) of cases, and moderate calcification was noted in three patients (3%).

**Table 4:** Coronary angiography findings in 100 patients

Findings	Number of patients	Percentage (%)
Lesion		
Type A	35	35%
Type B	15	15%
Type C	10	10%
Thrombus grade		
Grade 0	80	80%
Grade 2	6	6%
Grade 3	5	5%
Grade 4	5	5%
Grade 5	4	4%
Calcification		
Mild	7	7%
Moderate	3	3%

Table 5 presents the procedural interventions and management strategies for 55 patients (55%) undergoing PCI. Fibrinolysis was administered to 55 patients (55%), making it the most common treatment. Primary PCI was performed in 20 patients (20%), and pharmaco-invasive PCI was used in 10 patients (10%). Elective PCI accounted for 15 patients (15%) of procedures. Additionally, eight patients (8%) underwent CABG, while 40 patients (40%) received medical management as their primary treatment approach.

**Table 5:** PCI data of 55% of patients and their presentations CABG, coronary artery bypass graft; PCI, percutaneous coronary intervention

Findings	Count	Percentage (%)
Fibrinolysis	95	95%
Primary PCI	20	20%
Pharmaco-invasive PCI	10	10%
Elective PCI	15	15%
CABG	8	8%
Medical management	40	40%

Table 6 presents data for patients with nonobstructive CAD who underwent PCI. Specifically, seven patients (7%) had involvement in the LAD artery, five patients (5%) in the RCA, two patients (2%) exhibited anomalous origins of coronary arteries, and four patients (4%) had SCADs. Additionally, 32 patients (32%) had recanalized or normal coronary arteries.

**Table 6:** PCI patient data CAD, coronary artery disease; LAD, left anterior descending; RCA, right coronary artery; PCI, percutaneous coronary intervention; SCAD, spontaneous coronary artery dissection

Findings	Count (N)	Percentage (%)
Nonobstructive CAD		
LAD artery	7	7%
RCA	5	5%
Anomalous coronary artery origin	2	2%
SCAD	4	4%
Recanalized/normal coronary arteries	32	32%

## Discussion

CAD is the most prevalent form of heart disease and the primary cause of death both in the United States and globally. Accurate clinical assessment and coronary angiographic evaluation are essential for early detection and intervention to mitigate the disease’s associated morbidity and mortality [5,10,11]. This study demonstrates the clinical assessment and angiographic profiling of young STEMI patients from a tertiary care center, focusing on individuals aged ≤45 years. The study included 100 patients with a mean age of 42.5 years, of whom nine patients (9%) were under 25 years old and 89 patients (89%) were male. The identified risk factors for young CAD included smoking in 81 patients (81%), a history of premature CAD in 18 patients (18%), being overweight in 18 patients (18%), diabetes in 40 patients (40%), and hypertension in 29 patients (29%). The dyslipidemia pattern showed TC at 210.5 ± 35.50 mg/dL, LDL at 125.8 ± 20.40 mg/dL, TG at 195.3 ± 45.80 mg/dL, and HDL at 42.1 ± 6.20 mg/dL, indicating low HDL and high TG as characteristics of diabetic dyslipidemia [12].

Chest pain was the most commonly reported symptom of CAD, occurring in 99 patients (99%), while syncope was the least common, observed in 13 patients (13%) of cases. According to the clinical diagnosis, 39 patients (39%) had an inferior wall MI, four patients (4%) had an inferior wall MI combined with right ventricular MI, and three patients (3%) had a lateral wall MI. Of the patients, 60 patients (60%) had an anterior wall MI. The results of the coronary angiography showed that obstructive CAD was present in 65.9 patients (65.9%). Of these, 50 patients (50%) had single-vessel disease (SVD), nine patients (9%) had double-vessel disease, and 12 patients (12%) had triple-vessel disease. In five patients (5%), LM disease was discovered. The LAD artery was the most often damaged, appearing in 24 patients (24%), followed by the RCA in 14 patients (14%). Ostial LAD and ostial RCA were involved in two patients (2%) and four patients (4%) of cases, respectively.

Coronary angiography also indicated that Type A coronary lesions were present in 35 patients (35%), Type B in 15 patients (15%), and Type C in 10 patients (10%), suggesting a high success rate after treatment for type A lesions. Thrombus grades 0 to 5 showed that 80 patients (80%) had no thrombus characteristics. Coronary calcification was mild in seven patients (7%) and moderate in three patients (3%). Treatment profiling revealed that 55 patients (55%) underwent PCI, with 15 patients (15%) treated by elective PCI, 10 patients (10%) by pharmaco-invasive PCI, and 20 patients (20%) by primary PCI. Additionally, eight patients (8%) were referred for CABG.

It is interesting to note that 12 patients (12%) had nonobstructive CAD, with the LAD artery accounting for seven patients (7%) of cases and the RCA for five patients (5%). Additionally, 40 patients (40%) received medical care, and 32 patients (32%) had recanalized or normal coronaries. This research did not record any deaths. There are several publications on CAD patients’ clinical and coronary angiography results. In 117 young patients (less than 40 years old) having coronary angiography, Prakash et al. [2] recorded the angiographic and demographic profiles. They found that most affected young patients were male, with SVD in the LAD artery being the most common cause.

Similarly, Malakar et al. [4] reported the clinical and angiographic profile of CAD in 143 young women, showing that most patients had stable angina and STEMI, with hypertension and diabetes as common risk factors. Younger women were more affected than older women, according to their coronary angiography findings. This study adds substantial data on the clinical and coronary angiographic profiles of young patients with acute STEMI from a tertiary care center, aligning with the findings of Visnudas et al. [13]. Additionally, the correlation between the angiographic severity of CAD and glycemic status, as discussed by Muhammad et al. [14] and Parkar et al. [15], and the incidence and outcomes in younger patients, as highlighted by Jortveit et al. [16], further emphasize the relevance of glycemic control and demographic variations in the management of CAD.

Limitations of the study

Limited sample size is one of the study’s key shortcomings. Moreover, the study was conducted at a single site, which may limit the generalizability of the findings. Studying a larger patient group across various sites is crucial to gaining a more comprehensive understanding of the management and progression of the disease. Furthermore, long-term clinical and angiographic profiling of patients over several years is desirable to accurately estimate the actual mortality rate.

## Conclusions

In this cohort, very young males were the primary group associated with AMI, with smoking being the most prevalent risk factor. The most common cause of AWMI was the involvement of the LAD artery. Unlike the Western population, AWMI in this group presented later, exhibited greater severity, and was more diffuse, leading to higher morbidity despite favorable in-hospital mortality rates.
